# Synthesis and Structure of Vanadium–Lead Coatings for Outer Space Structures

**DOI:** 10.3390/ma17235785

**Published:** 2024-11-26

**Authors:** Yu. Zh. Tuleushev, V. N. Volodin, Y. A. Zhakanbayev, A. A. Migunova, B. M. Tynyshbay

**Affiliations:** Institute of Nuclear Physics, Almaty 050032, Kazakhstan; yuriy.tuleushev@inp.kz (Y.Z.T.); volodin@inp.kz (V.N.V.); zhakanbayev.yeldar@inp.kz (Y.A.Z.); a.migunova@inp.kz (A.A.M.)

**Keywords:** thin film coatings, X-ray diffraction, ion-plasma setup, vanadium–lead system

## Abstract

This study aimed to develop a technology for synthesizing non-degrading porous coatings that remain stable under the conditions of outer space. Using the magnetron sputtering method, we obtained vanadium–lead system coatings over a wide range of mutual concentrations from 6 to 44.9 at. % Pb. X-ray and electron microscopic studies were conducted on the obtained coatings. As a result of the research, a dependence of the film structure on changes in the lead concentration in vanadium was demonstrated. A correlation between the lead concentration in vanadium, the size of the resulting crystallites of solid solutions, and the change in the lattice parameter of the solid solution depending on the lead concentration in the coating was identified. A new possibility of producing porous vanadium coatings through vacuum annealing of the obtained alloy-based coatings in the V-Pb system was shown.

## 1. Introduction

Thermal-regulating coatings, both those that reflect radiation [1] and those that absorb it (the so-called “black”) [2,3,4], are of great importance for the functioning of artificial Earth satellites. Currently, the most commonly used light-absorbing coatings are enamels based on soot and acrylic resin; the main disadvantage of such coatings is their degradation in space conditions [5,6,7]. To create non-degradable “black” coatings that operate sustainably in outer space conditions, in our opinion, it is advisable to use coatings made of porous refractory metals along with ceramic materials [8]. In past research, we have shown that a porous tantalum coating can be obtained via the co-deposition of tantalum and cadmium, followed by the evaporation of cadmium in a vacuum when heated to temperatures above 700 °C [9]. In this regard, it is of interest to study the vanadium–lead system obtained by combined magnetron sputtering of components in alternating nanolayers and subsequent vacuum annealing.

Vanadium has a density of 6.11 g/cm^3^, a melting point of 1900 °C, and a boiling point of 3400 °C. Pure vanadium exhibits good ductility and can undergo various types of processing: forging, rolling, stamping, pressing, cutting, and welding in an inert atmosphere. It maintains its strength well at temperatures between 400 and 500 °C. Vanadium is minimally prone to work hardening and can withstand significant compression in a cold state without intermediate annealing.

To date, information about the state diagram, alloys, and compounds, as well as thermodynamic studies of the vanadium–lead system, has been poorly studied [10,11]. Data on the structure of any phases or compounds, apart from vanadium and lead, are absent in ICDD tables (The International Centre for Diffraction Data) and other reference publications.

The ion-plasma formation of materials using nanoparticle flows of sputtered elements, followed by the deposition of thin-film coatings on products, allows the creation of metal alloys with significantly different physical properties compared to materials obtained through traditional alloying technology. The process of magnetron alloy formation is based on the co-deposition of sputtered nanoparticles, which, according to the concept of thermofluctuational melting [12], are in a quasi-liquid state and capable of interacting during the deposition of two metals, leading to the formation (upon reaching a critical droplet size) of a solid solution (SS), typically with an increased concentration limit of its existence.

Given that vanadium, like lead, has a body-centered cubic (BCC) lattice, there is reason to believe that lead’s solubility in vanadium may increase when vanadium is alloyed with lead at the nanoscale. In our opinion, subsequent heat treatment may lead to the formation of a porous coating based on vanadium.

## 2. Experimental Details

**Figure 1:**
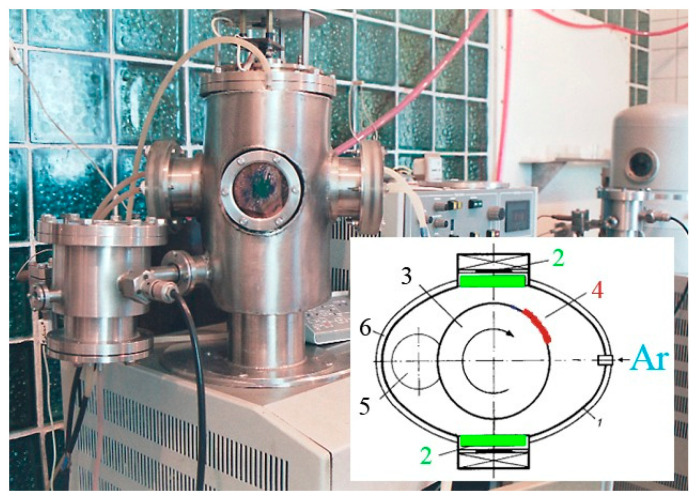
Schematic of the setup for forming coatings comprising vanadium–lead: 1—vacuum chamber body; 2—magnetrons; 3—cylinder; 4—substrate; 5—gas evacuation window; 6—caisson.

To prepare coating samples of tungsten lead, an ion-plasma system with two independent magnetron-sputtering devices was developed [12], the schematic of which is shown in Figure 1.

The setup consists of a vacuum chamber, on the walls of which planar direct-current (DC) magnetrons are mounted. The vacuum chamber was evacuated to a pressure of 3 × 10^−3^ Pa, and then ultra-pure argon was injected into the chamber to a pressure of 1 × 10^−1^ Pa, which is the working pressure for combustion of the magnetron device. The sputtering targets serve as cathodes, behind which water-cooled magnetic systems are located. Inside the vacuum chamber, a rotating cylinder is mounted to hold the substrates and move them relative to the magnetrons. A rotation speed of 10 revolutions per minute was selected to prepare vanadium–lead system coating samples. Thus, during the sputtering process, which lasted 40–60 min for coatings developed in this system, each substrate passed by the vanadium and lead sputtering magnetrons 400–600 times. The experimental setup is described in more detail in [12].

The sample preparation involved the sequential operations of simultaneous sputtering of vanadium and lead targets in a low-pressure plasma and the deposition of sputtered materials from vanadium and lead throughout the sputtering time.

The substrate temperature during sample formation did not exceed 100 °C. In the experiments, vanadium (99.96 wt.%) and lead (99.99 wt.%) were used as targets, with diameters of 40 mm and thicknesses of 4 mm.

The ratio of metal concentrations in the samples was adjusted by varying the sputtering rate of the planar magnetron sputtering targets. During the sputtering process, constant power was maintained on each sputtering device. The ratio of deposited components was controlled by weighing based on the amount of metal sputtered and deposited during the coating formation.

X-ray diffraction (XRD) measurements were conducted on a Bruker D8 “Discover” X-ray diffractometer. The X-ray beam, originating from a copper target, was collimated using a parabolic multilayer mirror and a pyrolytic graphite monochromator set to isolate Cu Kα radiation. The data were collected using Bragg–Brentano geometry (Θ–2Θ) over an angular range of 2Θ = 20–90° with a step size of 0.01°.

Electron microscopy studies were carried out using the JSM-8230 electron probe microanalyzer (JEOL, Tokyo, Japan). High-temperature annealing was performed in a high-temperature vacuum furnace designed based on the URVT-2500 system.

Measurements of the reflectivity of coatings were carried out on a Shimadzu (Kyoto, Japan) UV-1800 spectrophotometer by comparing the intensity of the reflected beam with respect to the reference beam.

## 3. Results

Using the dual-magnetron system, nine coatings of the V-Pb system were deposited, with the technological data and resulting structures presented in Table 1. The coatings were deposited on substrates made of α-Al_2_O_3_ with a thickness of 1 mm, glass with a thickness of 1.5 mm, and stainless steel foil 12Cr18Ni10Ti with a thickness of 0.20 mm.

The powers specified in the table, 1.0 W and 181 W, represent the lower and upper limits of the power regulation for the specialized power supply used in our magnetron setup.

**Figure 2:**
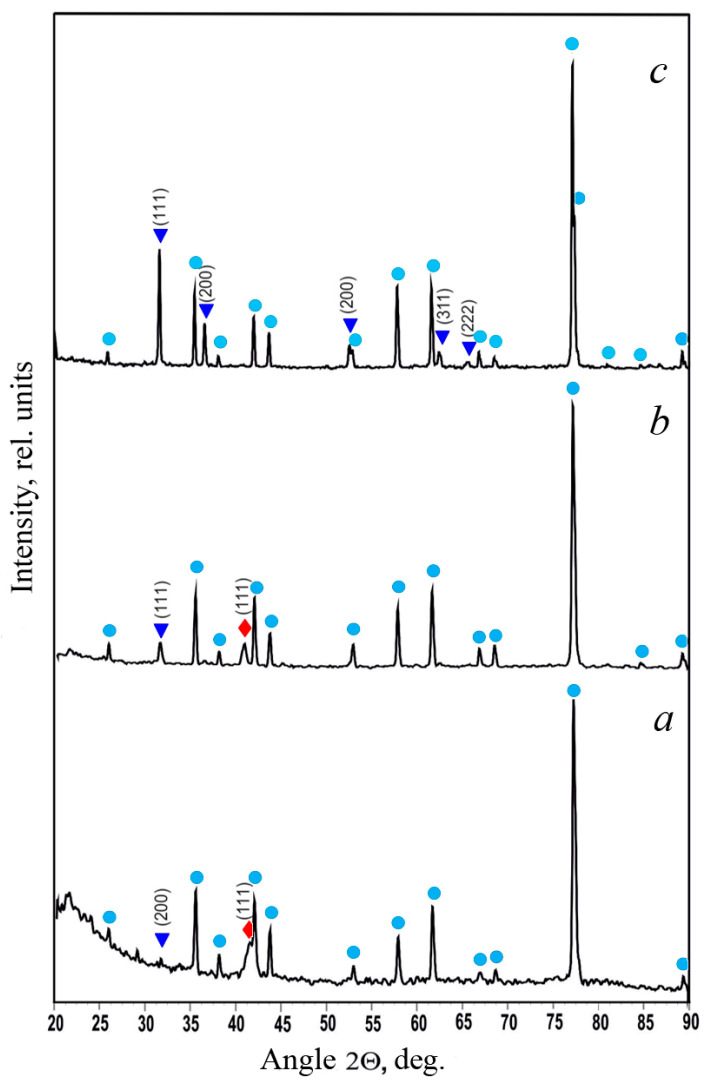
Diffractograms of V-Pb system coatings with a lead concentration of 6.0 at.% (**a**), 13.2 at.% (**b**), and 24.9 at.% (**c**) sputtered on α-Al_2_O_3_ substrates; ●—α-Al_2_O_3_, ▼—solid solution of V in Pb, ⧫—solid solution of Pb in V.

X-ray diffraction studies were conducted on all obtained coatings. Figure 2 displays the diffractograms of the V-Pb system coatings with varying lead concentrations.

The diffractogram analysis indicates that at a lead concentration in the coating greater than 24.9 at.%, only the solid solution of vanadium in lead is detected, with no vanadium even appearing in an amorphous state. However, this does not mean that all vanadium dissolves in lead. The reflective ability of lead atoms is approximately ten times higher than that of vanadium, which should be taken into account. Considering that the lattice parameter of the detected lead phase of 0.4948 nm significantly exceeds the tabulated lattice parameter of lead, *a*_Pb_ = 0.4930 nm (PDF№ 87-0663), it can be concluded that some vanadium is dissolved in the lead, although the exact amount cannot be determined from the diffractogram analysis.

At a lead concentration in the coating of 13.2 at.%, reflections from both the solid solution phase of vanadium in lead with a lattice parameter a = 0.4954 ± 0.0003 nm (PDF№ 87-0663, reflection (111)) and the solid solution phase of lead in vanadium with a lattice parameter a = 0.3847 nm (PDF№ 88-2322) are detected simultaneously. At a lead concentration in the coating of 6.0 at.%, the phase of the solid solution of lead in vanadium with a lattice parameter a = 0.3772 nm is detected (PDF№ 88-2322), and there are indications of the existence of the solid solution phase of vanadium in lead with a lattice parameter a = 0.4949 nm (PDF№ 87-0663, reflection (200)). Therefore, throughout the observed range of lead concentrations in the coating, both the solid solution of lead in vanadium and the solid solution of vanadium in lead coexist, with the latter becoming unobservable at higher lead concentrations (>24.9 at.% Pb) due to the amorphization of this phase and its low reflective ability compared to lead.

**Figure 3:**
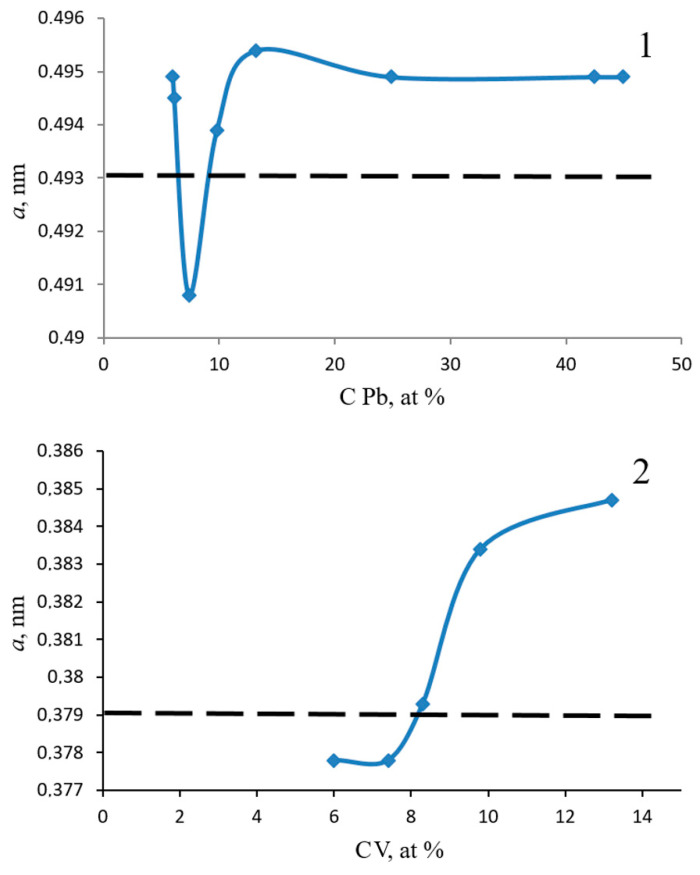
Graph of dependence of the lattice parameter of vanadium solid solution in lead on the lead concentration in the coating. The dotted line in picture 1 shows the tabulated lattice parameter of lead a = 0.4930 nm (PDF№ 87-0663). The dotted line in picture 2 shows the tabulated lattice parameter of vanadium a = 0.3790 nm (PDF№ 88-2322).

Figure 3 shows the graph of dependence of the lattice parameter of the vanadium in lead (1) and lead in vanadium (2) solid solution on the concentration of lead in the coating.

The phenomenon of lattice parameter inversion with changing concentrations of alloying impurity has never been observed in the study of solid solutions.

**Figure 4:**
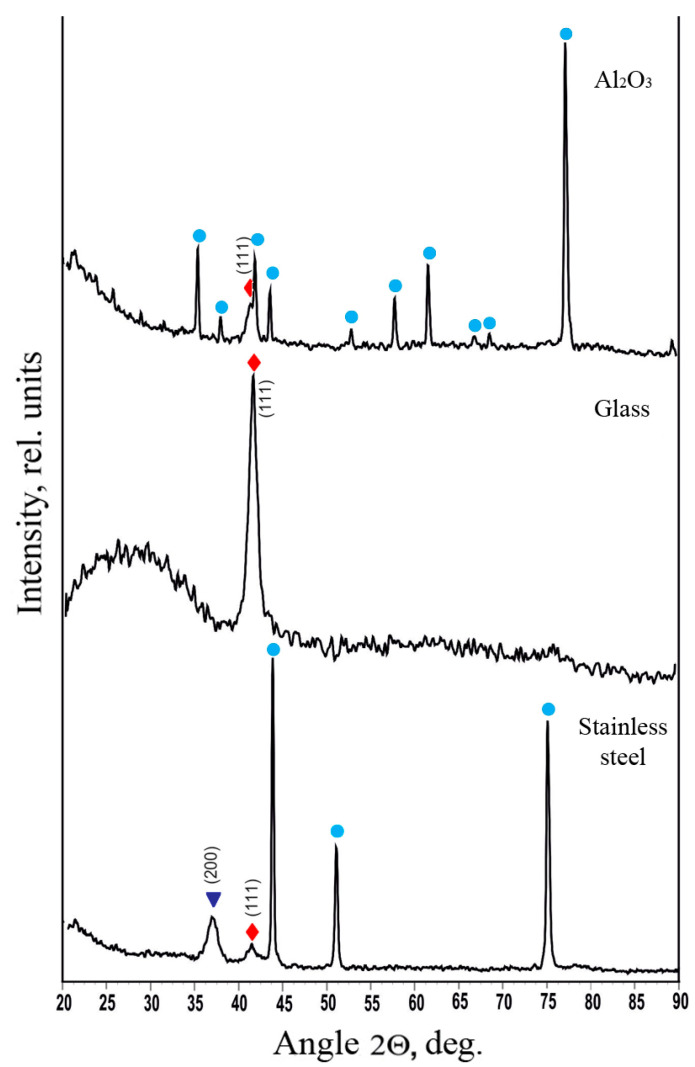
Diffractograms of V-Pb system coatings with a lead concentration of 6.0 at.% sputtered on substrates of α-Al_2_O_3_, glass, and stainless steel; ●—α-Al_2_O_3_; ▼—solid solution of V in Pb, ⧫—solid solution of Pb in V.

It is also important to note the dependence of the crystallization of solid solution phases of lead in vanadium and vanadium in lead on the type of substrate used for the deposition. Figure 4 shows the diffraction patterns of coatings with a lead concentration of 6.0 at.% deposited on α-Al_2_O_3_, glass, and stainless steel foil 12Cr18Ni10Ti substrates.

Given that the various substrates were under identical technological conditions during the deposition (the distance from the sputtering magnetrons, time spent in the metal-containing plasma stream, and total deposition time), it can be assumed that the crystallization processes of the solid solution phases are influenced by the thermal conductivity of the substrates. Glass has the lowest thermal conductivity, α-Al_2_O_3_ is higher, and stainless steel 12Cr18Ni10Ti has the highest.

To explain the anomalies in the lattice parameter dependence of the lead solid solution in vanadium on the lead concentration in the coating at low lead concentrations, we considered the change in the crystallite sizes of the solid solutions (SS) of vanadium in lead (V in Pb SS) and lead in vanadium (Pb in V SS), calculated from the broadening of diffraction peaks using the Scherrer formula and Eva software integrated into the Bruker diffractometer software. For the coating with a 6.0 at.% Pb concentration deposited on an α-Al_2_O_3_ substrate (Figure 4), the crystallite size of V in Pb SS, calculated from the (200) Pb peak, is 5.3 nm, while the crystallite size of Pb in V SS, calculated from the (111) V peak, is 10.7 nm. When deposited on a glass substrate (Figure 4), the coating with a 6.0 at.% Pb concentration has a Pb in V SS crystallite size of 8.54 nm, calculated from the (111) V peak. When deposited on a stainless steel substrate (Figure 4), the crystallite size of V in Pb SS, calculated from the (200) Pb peak, is 7.2 nm, and the crystallite size of Pb in V SS, calculated from the (111) V peak, is 21.2 nm. Thus, it is evident that the solid solutions of lead in vanadium and vanadium in lead crystallize into very small grains within this concentration range. The influence of grain boundaries on the lattice parameter of the obtained solid solutions explains the observed anomalies in the lattice parameter dependence of the vanadium solid solution in lead on the lead concentration in the coating, as well as the lattice parameter of the lead solid solution in vanadium at Pb concentrations of 6 ÷ 10 at.%.

To explore the possibility of obtaining porous structures from vanadium and assess the effect of lead content on the results, vacuum annealing was performed at 800 °C for 1 h on two V-Pb system coatings with lead contents of 24.9 and 44.9 at.%, deposited on α-Al_2_O_3_ substrates. Initially, after deposition, they represent a solid solution of vanadium in lead (for the 24.9 at.% Pb coating) and nearly pure lead (for the 44.9 at.% Pb coating). After annealing at 800 °C for 1 h, only one reflection with d_hkl_ = 0.2054 nm was observed in the 24.9 at.% Pb coating, which cannot be attributed to either V in Pb SS or Pb in V SS. This is likely a new intermetallic phase in the V-Pb system, but with only one reflection. Neither the symmetry of the assumed phase nor its lattice parameter can be determined. The 44.9 at.% Pb coating became completely X-ray amorphous after vacuum annealing.

**Figure 5:**
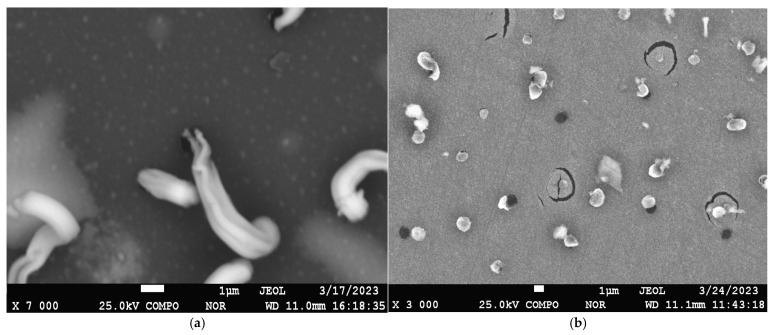
SEM image of the surface of the sample of the coating of the vanadium–lead system with lead content of 24.9 at.% before (**a**) and after (**b**) vacuum annealing at 800 °C for 1 h.

Figure 5 shows the results of SEM studies of the coating with a lead content of 24.9 at.% sputtered on α-Al_2_O_3_ substrates before and after vacuum annealing at 800 °C for 1 h.

In the pre-annealing surface image, a fine-grained structure is visible, from which lead whiskers grow in a twisted manner, similar to how the cream is squeezed out of a tube, as observed in [11]. After annealing, deep cracks and pores appear on the surface in the locations where the whiskers protruded, and only thin shell-like remnants of the whiskers in the form of petals remain. According to EDS mapping data of the sample surface after annealing, the coating surface consists solely of vanadium.

**Figure 6:**
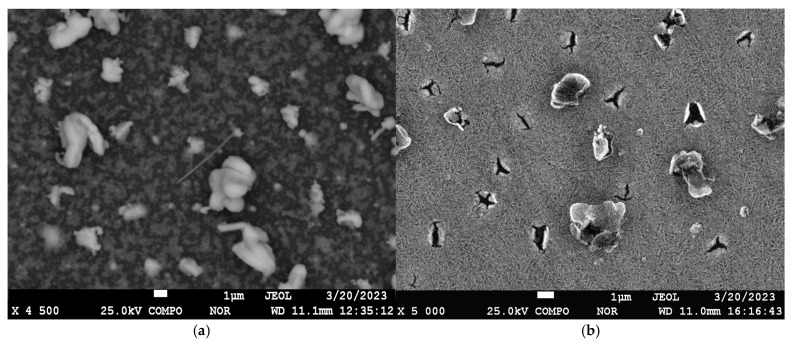
SEM image of the surface of the sample of the coating of the vanadium–lead system with lead content of 44.9 at.% before (**a**) and after (**b**) vacuum annealing at 800 °C for 1 h.

Figure 6 shows the results of SEM studies of the coating with a lead content of 44.9 at.% sputtered on α-Al_2_O_3_ substrates before and after vacuum annealing at 800 °C for 1 h.

In the pre-annealing surface image, a fine-grained structure is visible, from which lead whiskers also grow, but in this case, they twist into a dense bundle. After annealing, the number of cracks increases, and the remnants of the whiskers appear as thin shell-like structures in the form of petals. According to EDS mapping data of the sample surface after annealing, the coating surface consists solely of vanadium.

**Figure 7:**
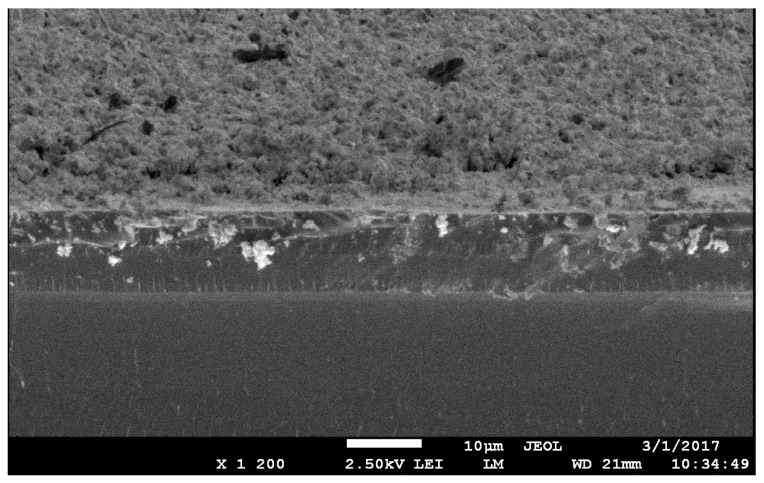
SEM image of the broken surface of a sample of a porous coating of a vanadium–lead system with a lead content of 44.9 at.% after vacuum annealing at 800 °C for 1 h.

Figure 7 shows the fracture of a porous vanadium coating deposited on a silicon substrate after heat treatment at 800 °C for 1 h. It can be seen that the coatings are fine-celled metal foam.

Thus, it has demonstrated that the co-deposition of vanadium and lead from two oppositely positioned magnetrons onto moving substrates results in solid solutions of lead in vanadium and vanadium in lead. The obtained solid solutions of Pb in V are thermally unstable in a vacuum: at T = 800 °C, lead diffusion to the surface and evaporation of lead into the vacuum was observed.

The resulting porous coatings were tested on a Shimadzu UV-1800 spectrophotometer and showed a reflectance coefficient of 4–5% (emissivity coefficient 95–96%). To test the temporal stability, samples of porous coatings were sent to the ISS (International Space Station).

## 4. Conclusions

Using magnetron sputtering, coatings of the vanadium–lead system were obtained in a wide range of mutual concentrations, from 6 to 44.9 at.%. X-ray diffraction and electron microscopy studies were carried out to describe the processes of structural and phase transformations when the concentration of lead in vanadium changes. It was shown that in films with a lead concentration of more than 9.8 at.%, excess lead is released into a separate phase with lattice parameters close to those of pure lead. There is a correlation between the concentration of lead in vanadium and the size of crystallites in solid solutions of vanadium in lead and lead in vanadium. An anomaly was found in the behavior of the dependence of the lattice parameter of the solid solution of lead in vanadium with an increase in the concentration of lead in the coating. This can be explained by the fact that at low lead concentrations, the crystallite size changes in the solid solutions of vanadium in lead (V in Pb) and lead in vanadium (Pb in V). SEM/EDS studies revealed the presence of lead whiskers at lead concentrations of 24.9 and 44.9 at.%, indicating the segregation of excess lead from the coating. Further heat treatment in vacuum at 800 °C for one hour resulted in almost complete removal of lead from the coating, resulting in the formation of a porous coating structure, which was confirmed by the results of X-ray diffraction and EDS mapping. The resulting porous coatings showed a reflectivity of 4–5% (emissivity of 95–96%).

## Figures and Tables

**Table 1:** Power in the vanadium (W_V_) and lead (W_Pb_) sputtering channels, the amount of vanadium (n_V_) and lead (n_Pb_) sputtered in one cycle, partial thicknesses of the vanadium (d_V_) and lead (d_Pb_) layers deposited per revolution, total coating thickness (d), calculated lead concentration in the coating (C_P_), and the structure of the phases obtained from the deposition.

W_V_, Watt	W_Pb_, Watt	n_V_, mmol	n_Pb_, mmol	d_V_ nm/Layer	d_Pb_, nm/Layer	Thickness, µm	C_Pb_, at.%	Structure (Substrate)
181	1.0	3.361	0.214	0.78	0.110	268	6.0	V *a* = 0.3772 nm (α-Al_2_O_3_) Pb *a* = 0.4949 nm (α-Al_2_O_3_) V *a* = 0.3771 nm (glass) Pb-V *a* = 0.3765 nm (steel) Pb *a* = 0.4884 nm (steel)
181	1.0	6.478	0.410	0.76	0.110	513	6.0	V *a* = 0.3793 nm (α-Al_2_O_3_) Pb *a* = 0.4945 nm (α-Al_2_O_3_) V *a* = 0.3783 nm (glass) V *a* = 0.3782 nm (steel) Pb *a* = 0.4866 nm (steel)
141	1.0	5.087	0.405	0.60	0.100	417	7.4	V *a* = 0.3774 nm (α-Al_2_O_3_) V *a* = 0.3779 nm (glass) V *a* = 0.3774 nm (steel) Pb *a* = 0.4908 nm (steel) a = 0.3783 nm (c-Si)
140	1.0	3.057	0.275	0.61	0.112	256	8.3	V *a* = 0.3793
113.3	1.0	3.152	0.343	0.49	0.120	273	9.8	V *a* = 0.3835 nm (α-Al_2_O_3_) Pb *a* = 0.4939 nm (α-Al_2_O_3_) V *a* = 0.3833 nm (glass) Pb *a* = 0.4952 nm (glass)
100	1.0	2.483	0.376	0.44	0.140	230	13.2	Pb *a* = 0.4954 nm + V *a* = 0.3847 nm (α-Al_2_O_3_, glass, steel)
50.	2.0	2.599	0.864	0.22	0.170	314	24.9	Pb *a* = 0.4949 nm (α-Al_2_O_3_, glass, steel)
50	4.6	1.930	1.419	0.21	0.330	352	42.4	Pb *a* = 0.4949 nm (α-Al_2_O_3_, glass, steel)
50	6.0	1.770	1.442	0.21	0.480	414	44.9	Pb *a* = 0.4948 nm (α-Al_2_O_3_, glass, steel)

## Data Availability

The original contributions presented in the study are included in the article: further inquiries can be directed to the corresponding author.
